# A new strategy: identification of specific antibodies for neutralizing epitope on SARS-CoV-2 S protein by LC-MS/MS combined with immune repertoire

**DOI:** 10.1186/s43556-022-00085-0

**Published:** 2022-07-05

**Authors:** Meng Yu, Zhu Zhu, Yanqun Wang, Pingzhang Wang, Xiaodong Jia, Jie Wang, Lei Liu, Wanbing Liu, Yaqiong Zheng, Guomei Kou, Weiyan Xu, Jing Huang, Fengmin Lu, Xiajuan Zou, Shangen Zheng, Yinying Lu, Jincun Zhao, Hui Dai, Xiaoyan Qiu

**Affiliations:** 1 Department of Immunology, School of Basic Medical Sciences, Peking University, and NHC Key Laboratory of Medical Immunology (Peking University), Beijing, China; 2 Key Laboratory of Molecular Immunology, Chinese Academy of Medical Sciences, Beijing, China; 3 State Key Laboratory of Respiratory Disease, National Clinical Research Center for Respiratory Disease, Guangzhou Institute of Respiratory Health, the First Affiliated Hospital of Guangzhou Medical University, Guangzhou, Guangdong China; 4 Department of Hepatology, Fifth Medical Center of Chinese PLA General Hospital, Beijing, China; 5 Department of Microbiology and Infectious Disease Center, School of Basic Medical Sciences, Peking University Health Science Center, Beijing, China; 6 Department of Transfusion Medicine, General Hospital of Central Theater Command of PLA, Wuhan, Hubei China; 7 Medical and Healthy Analysis Center, Peking University, Beijing, China

**Keywords:** COVID-19, Neutralizing antibody, Neutralizing epitope;LC-MS/MS

## Abstract

Although the SARS-CoV-2 vaccine has been widely used worldwide, not all individuals can produce neutralization antibodies, so it is still urgent to find and prepare neutralization antibodies for COVID-19 prevention or treatment. In this study, we created a new strategy to effectively obtain neutralizing antibodies or complementary determining region 3 (CDR3) of neutralizing antibodies against SARS-CoV-2. We first predicted and synthesized several B cell epitopes on RBD and adjacent RBD of S protein, then the B cell epitopes were used to prepare affinity chromatography columns respectively and purify the binding IgG from serum samples of convalescent COVID-19 patients. After these IgGs were identified to have neutralizing activity, the peptide sequences of the antigen-binding regions (variable region) of neutralizing antibodies were analyzed by protein mass spectrometry. Subsequently, the B cells from the same individual were sorted and used to obtain their full BCR repertoire by 5′ RACE combined with high-throughput of PacBio sequencing method. Then, the peptide sequence of neutralizing antibody variable region by protein mass spectrometry was mapped to the full BCR repertoire and found the full variable region sequence of neutralizing antibodies. Finally, we obtained and synthesized numerous CDR3 peptides of neutralizing antibodies to confirm the neutralizing activity for SARS-CoV-2 infection. Our results indicate that the novel scheme will be suitable for rapid screening of neutralizing antibodies, including screening neutralizing antibodies against SARS-CoV-2 and other pathogenic microorganisms.

**Supplementary Information:**

The online version contains supplementary material available at 10.1186/s43556-022-00085-0.

## Introduction

The coronavirus disease 2019 (COVID-19) pandemic caused by severe acute respiratory syndrome coronavirus 2 (SARS-CoV-2) presents a global health emergency that is in urgent need of intervention, which has caused millions of deaths (https://covid19.who.int/). Especially after the emergence of Delta(δ) and Omicron variants (https://outbreak.info/situation-reports), which challenges the effectiveness of the original SARS-CoV-2 vaccine that has been developed and vaccinated all over the world and make the outcome of the epidemic more difficult to predict [1, 2]. The entry of SARS-CoV-2 into its target cells depends on binding between the receptor-binding domain (RBD) of the viral spike (S) protein and its receptor, angiotensin-converting enzyme 2 (ACE2) [3]. Thereby, the RBD of the viral S protein is a key target for intervention of SARS-CoV-2 infection. It was demonstrated that neutralizing antibodies induced by RBD of S protein can block the binding of SARS-CoV-2 to the receptor [4–6] and protect the body from SARS-CoV-2 reinfection. Currently, the SARS-CoV-2 vaccine has been used worldwide, the protective effect of the vaccine for COVID-19 has been evaluated by clinical data. However, in fact, neither all vaccinated individuals or all naturally infected patients can produce protective antibodies [7, 8]. Moreover, vaccine development is slower than the SARS-CoV-2 mutation [9]. Therefore, it is urgent to develop and obtain effective antibodies or inhibitors against the SARS-CoV-2 virus to block the global epidemic of COVID-19.

The antibody is a “natural weapon” produced by immune cells after being stimulated by viruses and other antigens. It plays an irreplaceable role in the long history of the game between human and infectious diseases. Generally, when the virus enters the body, B cells can recognize the viral antigen through the B cell receptor (BCR) on its surface, then they are activated and clonal proliferated with the help of CD4^+^ T cells. Then, some B cells differentiate into antibody-producing cells (plasma cells) and produce a large number of antibodies to block the virus infection. While some B cells stop differentiation and turn into memory B cells to prepare for the second attack of the same antigen. It is worth noting that as long as they are differentiated from the same native B cell, both the BCR (on effector B cells and memory B cells) and the antibodies have identical antigen-recognition ability, which is dependent on their identical antigen-binding region (variable region).

To obtain the variable region sequence of SARS-COV-2 S protein-specific antibody, at present, scientists usually use S protein or its RBD sequence to screen the B cells that can bind to S protein or RBD, and then analyze its BCR sequence by single-cell sequencing or multiplex PCR technology. Next, the recombinant antibody repertoire was established using the BCR/antibody repertoire of variable region sequence and screen the antibodies with neutralizing ability [10].

To date, although several effective neutralizing antibodies have been identified using the scheme as above [11, 12], we have to say that current reported strategies for obtaining the variable region sequence of SARS-CoV-2 neutralizing antibodies need to be improved. According to the current reports, almost all of the patients with COVID-19 and vaccinated individuals can produce different titers of SARS-CoV-2 specific antibodies, but not all individuals produce the neutralizing antibodies. In other words, S protein, as well as its RBD of SARS-CoV-2, contains multiple antigen epitopes, but only a few antigen epitopes can induce neutralizing antibodies to block SARS-CoV-2 reinfection, which means that most BCRs that can bind to S protein or RBD have no neutralizing activity [13]. In addition, The genome of SARS-CoV-2 is relatively unstable compared to other RNA viruses which will lead to constant creation of new variants. The vaccines developed based on wild-type SARS-CoV-2 are less effective in preventing variant infections [8]. This may due to the epitopes that induce neutralizing antibody against wild-type virus were mutated so that the neutralizing antibody cannot effectively neutralize the variant. So only depend on the BCR repertoire to screen SARS-CoV-2 neutralizing antibody is uneconomic and inefficient and relative conservative epitope with neutralizing ability need to be discovery. Moreover, although the single-cell sequencing technology was widely used to analyze BCR repertoire, only dominant sequence can be detected due to limitation in sequencing depth and length by 10xGenome sequencing. In addition, to date, using the universal primers for multiple variable regions of Ig gene and multiple PCR method to amplify BCR repertoire, however, which usually show biased amplification instead of equal opportunity amplification for all variable regions.

In this study, we created a new strategy to effectively obtain neutralizing antibodies or its CDR3 against SARS-CoV-2. We first predicted the B cell epitopes in RBD and RBD vicinity of S protein by bioinformatics methods. Then we selected and synthesized four B cell epitope peptides, which were used to prepare affinity chromatography columns, respectively, and purified the antibodies from 15 serum samples of COVID-19 patients 2 weeks after recovery. After these antibodies were identified to have neutralizing activity, the antibody was then analyzed by protein mass spectrometry to obtain the peptide sequence. Subsequently, we sorted the B cells from the 15 COVID-19 patients 2 weeks after recovery and analyzed their BCR repertoire. It is worth noting that in order to avoid the bias amplification caused by multiple PCR and the insufficient BCR reads obtained by 10x Genomics or Sanger sequencing, we used 5′-RACE combined with high-throughput of PacBio sequencing method to obtain an unbiased, sufficient number of BCRs containing full-length variable region sequences. Then, the peptide sequence of neutralizing antibody variable region as described above was mapped to the BCR repertoire and found the gene sequence of neutralizing antibody variable region. Finally, we synthesized the CDR3 peptide of neutralizing antibodies or prepared to confirm the feasibility of the scheme.

## Results

### A novel strategy to obtain the specific sequencing of neutralizing antibodies for the neutralizing epitope on S protein

It is well known that only the antigen epitopes on RBD or on its neighborhood are critical for producing neutralizing antibodies that can directly block viruses to bind and invade cells. In this study, we have designed a new strategy to obtain the specific sequencing of neutralizing antibodies for RBD and its adjacent domain of S protein, which combining with the information obtained from protein mass spectrometry and BCR transcriptome analysis of neutralizing antibodies.

**Fig. 1:**
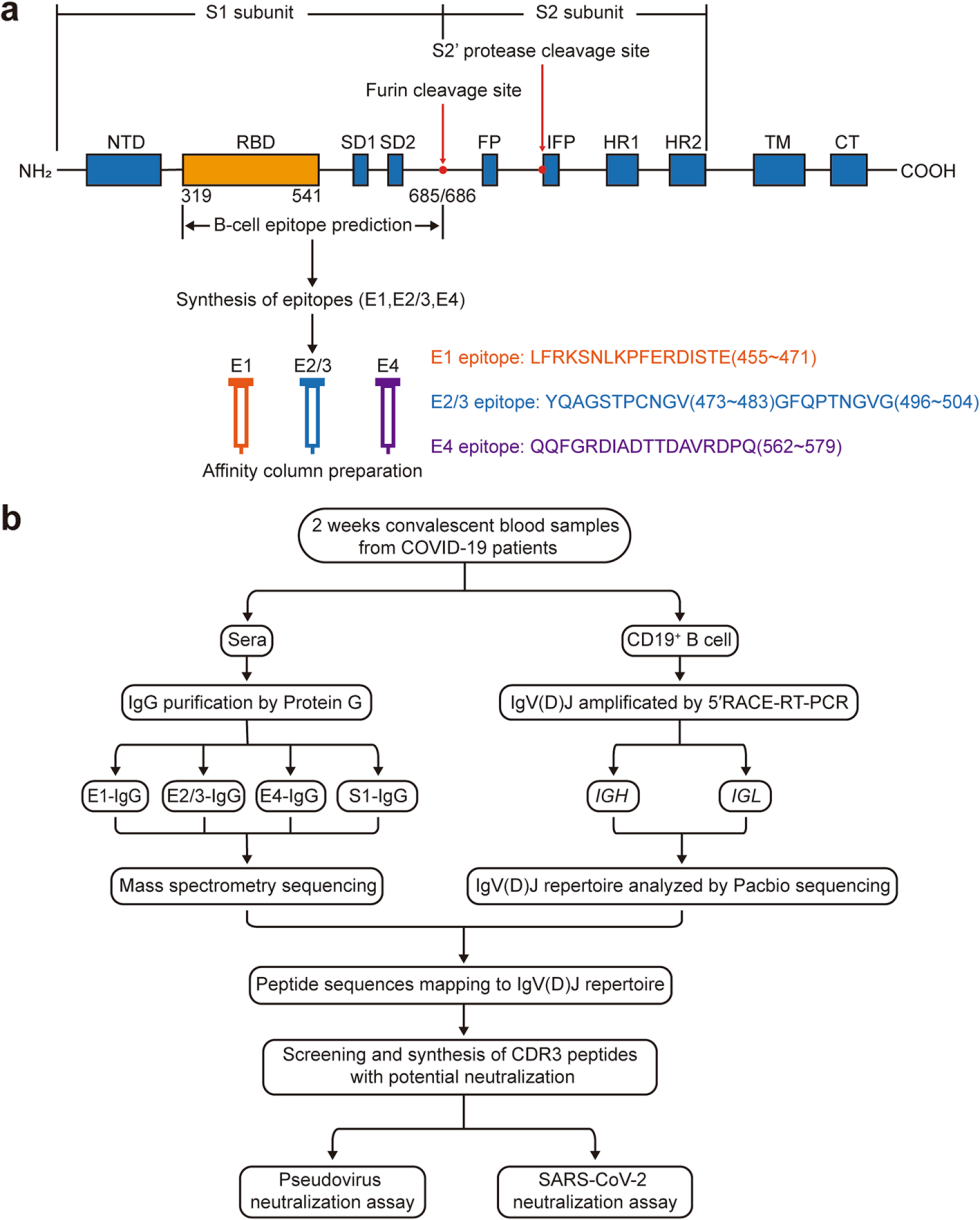
Schematic diagram of obtain CDR3 of neutralizing antibodies against SARS-CoV-2. **a** The spike protein (S protein) of SARS-CoV-2 comprises two functional subunits: S1 subunit can bind to the host cell receptor ACE2 and S2 subunit mediated the fusion of the viral and host cell membranes. This S1/S2 boundary constitutes the cleavage site for the subtilisin-like host cell protease furin, which is ubiquitously expressed in humans. The distal region of S1 subunit comprises RBDs and stabilizes the prefusion state of the membrane-anchored S2 subunit which contains the fusion machinery. Four linear B cell epitopes are located on the receptor-binding domain (RBD) or adjacent RBD. Then, the epitope peptides were synthesized and coupled to CNBr-activated Sepharose 4FF to purify epitope peptide-specific IgG. NTD, N-terminal domain; RBD, receptor-binding domain; SD1, subdomain 1; SD2, subdomain 2; FP, fusion peptide; IFP, internal fusion peptide; HR1, heptad repeat 1; HR2, heptad repeat 2; TM, transmembrane domain; IC, intracellular tail. **b** This flowchart shows the detailed steps of the entire scheme

In brief, firstly, the B cell antigen epitopes of RBD and its adjacent regions were predicted by the bioinformatics method, and four candidate epitopes were selected to synthesize and prepare the affinity chromatography column (Fig. 1a). The IgG binding to different epitopes was purified from the serum of convalescent COVID-19 patients (patient information was shown in Supplementary Table 1) by using these affinity chromatography columns, the block activity of IgG for AAV containing S protein infects to ACE2-expressed HEK293 cells was identified. Then, the IgG was used to analyze its peptide information of variable region sequence by protein mass spectrometry. However, only based on the current mass spectrometry analysis technology, the intact variable region sequence of IgG cannot be obtained. Thereby, we further sorted B cells from these COVID-19 patients to obtain the full variable region sequence of IgG by immune repertoire technology. After that, the above IgG variable region sequence obtained by protein mass spectrometry was mapped with variable region sequence among the BCR repertoire to obtain the whole gene sequence of IgG variable region, then the neutralizing activation of CDR3 of IgG variable region was identified. It is worth noting that, in order to maximize obtain the total BCR transcripts of COVID-19 patients, the BCR amplification method we choose is not the current multiple PCR, but the 5′-RACE method, which can avoid the biased amplification brought by the multiple PCR. In addition, the high throughput PacBio sequencing scheme is used to avoid the shortcomings of BCR information obtained by the current commonly used 10x Genomics and Sanger sequencing (Fig. 1).

### Identification of neutralization ability of IgG from the serum of COVID-19 patients of 2 weeks convalescence

**Fig. 2:**
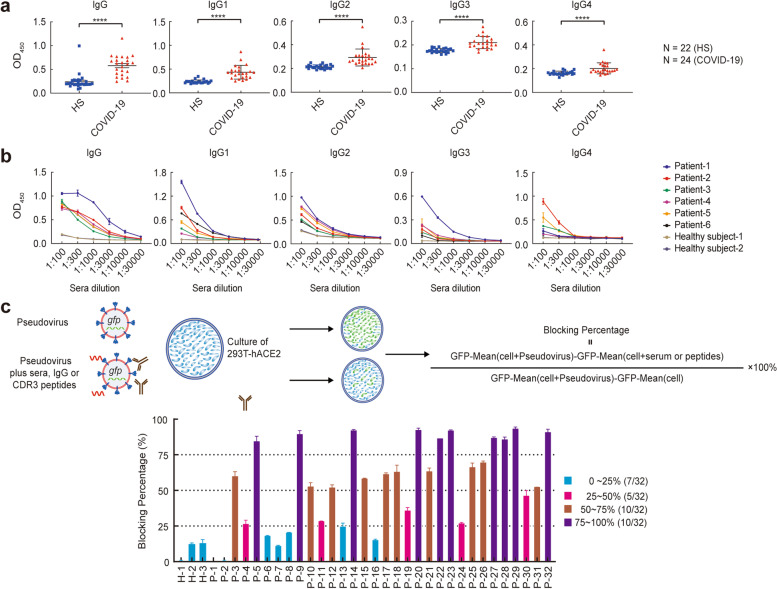
Neutralization ability of IgG in sera from COVID-19 patients of 2 weeks convalescence. **a** The sera from healthy subjects (HS) and COVID-19 convalescent were diluted 100x and detected S1 protein-specific IgG1, IgG2, IgG3, IgG4, and total S1 protein-specific IgG by ELISA. A microplate reader measured the absorbance at 450 nm. **b** Diluted sera (from 1:100 to 1:30000) were detected by ELISA to assure the S1 protein-specific IgG, IgG1, IgG2, IgG3, and IgG4. A microplate reader measured the absorbance at 450 nm. **c** The pseudovirus neutralization assays were performed using hACE2-expressed HEK293 cell lines. SARS-CoV-2 pseudoviruses containing GFP encoding gene were mixed with CDR3 peptides, purified IgG, and diluted sera were added to the well. After 18 hours of cell culture, the GFP-FITC was detected by flow cytometry. The neutralization efficiency of peptides/IgG/sera against SARS-CoV-2 pseudovirus was determined as [(Positive control MFI- experimental MFI)/(Positive control MFI- Negative control MFI)] × 100. d. The sera from 3 healthy subjects (HS) and 32 COVID-19 convalescents were diluted 160x and detected their ability to block pseudovirus by the method described above

IgG from the sera of 32 patients with COVID-19 have purified by protein G, and the SARS-CoV-2-S protein-specific IgG, including IgG1, IgG2, IgG3, IgG4, were also detected by ELISA. The results showed that compared with three healthy controls, all the COVID-19 patients displayed higher levels of SARS-CoV-2-S protein-specific antibodies, which showed a higher level about 30–100 times than that of healthy donors (Fig. 2a,b). To verify the neutralization ability of the IgG to the SARS-CoV-2 infection in serum of patients, we first established a pseudovirus infection model that using an AAV containing S protein-GFP of SARS-CoV-2 to infect the ACE2-expressed HEK293, then the neutralizing activation of the IgG from the serum of patients in the convalescence of 2 weeks was detected by flow cytometry, and found that although all the individuals analyzed in this study could detect S1 protein-specific antibodies about 25% of the individuals could not detect the neutralizing antibodies (more than 50% inhibition rate) under 10 μg/ml of IgG condition (Fig. 2c). These data indicated that almost all COVID-19 convalescent patients can produce specific antibodies against S protein of SARS-CoV-2, but only a few can produce neutralizing antibodies.

### Prediction and identification of neutralizing epitopes and neutralizing antibodies of S1 protein

**Fig. 3:**
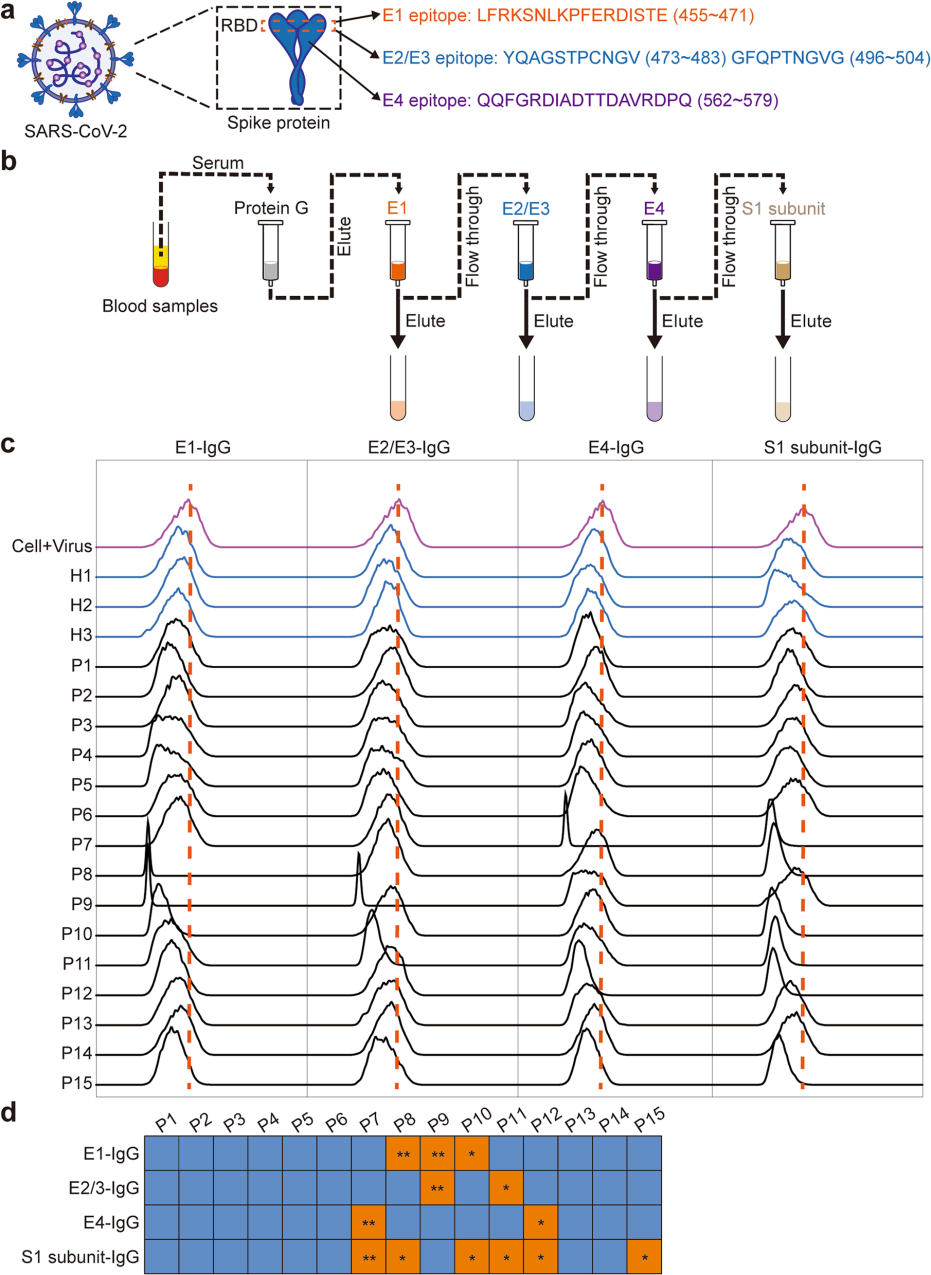
Neutralization activity analysis of purified antibodies on infection of SARS-CoV-2 pseudovirus to ACE2^+^ HEK293. **a** Four linear B cell epitopes, including LFRKSNLKPFERDISTE (455–47, E1), YQAGSTPCNGV (473–483, E2), GFQPTNGVGY (496–505, E3), and QQFGRDIADTTDAVRDPQ (563–580, E4). E1, E2, E3 are located on the receptor-binding domain (RBD), E4 is located on adjacent RBD. **b** The epitope peptides were synthesized and coupled to CNBr-activated Sepharose 4FF to purify epitope peptide-specific IgG. **c** The sera from 3 healthy subjects (HS) and 15 COVID-19 convalescents were diluted 160x and detected their ability to block pseudovirus by flow cytometry. GFP fluorescence intensity was compared. **d** The blocking efficiency of epitope peptide-specific IgG. Blue cells represent for no blocking effect (Blocking percentage < 50%?) while orange cells represent for have blocking effect. Blocking percentage(%):*45%–90%, **90%–100%

In order to find neutralizing antibodies that can recognize neutralizing epitope of S protein, we first used bioinformatics to predict the B cell epitopes on RBD and adjacent RBD regions and found four candidate B cell epitopes. Moreover, the Four B cell epitopes have been identified to be neutralizing epitopes of SARS. Thereby, we synthesized three epitope peptides, which are named E1, E2-E3, and E4, respectively. The E1 contained one predicted epitope (LFRKSNLKPFERIDITE) in RBD; E2-E3 contained two predicted epitopes (YQAGSTPCNGV and GFQPTNGVG) in RBD; E4 contained one predicted epitope (QQFGRDIADTAVDRDPQ), which located in RBD neighbor domain SD1 (Fig. 3a). It is worth noting that we have also analyzed the mutated SARS-CoV-2 gene sequence in Omicron variant. Clearly, we only found that there were five amino acid mutation in E2, and there was no mutation in above other candidate epitopes (Supplementary Table 3). The finding suggests that these candidate epitopes are conservative sequences of SARS-CoV-2, and the neutralizing antibodies against these epitopes can also block the infection of SARS-CoV-2 variants.

**Fig. 4:**
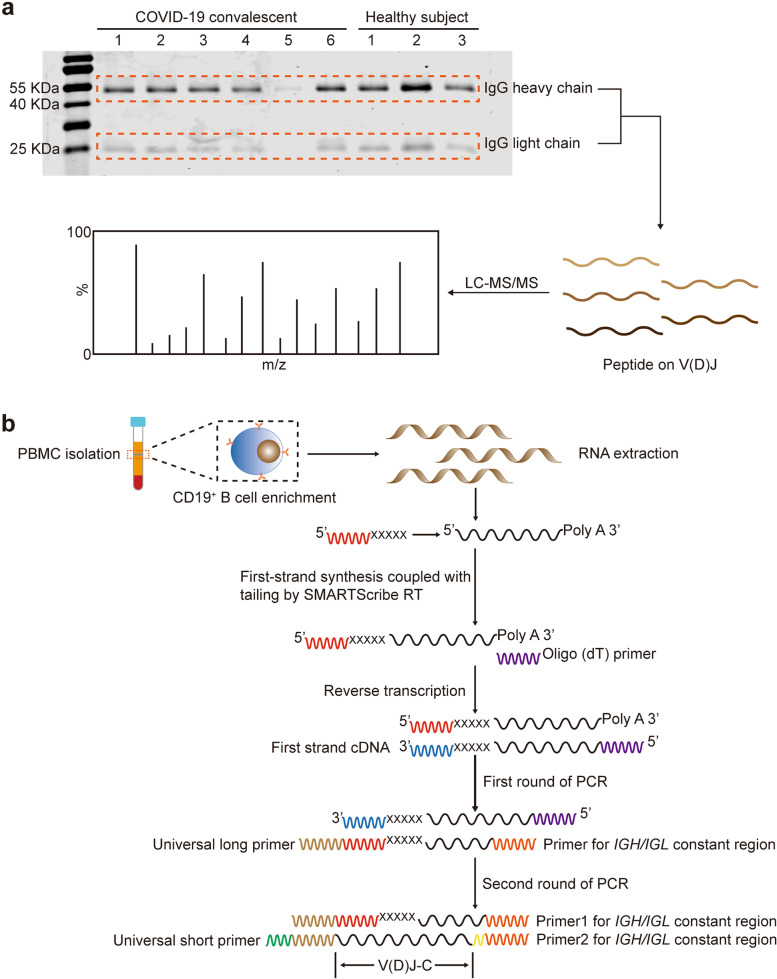
Mass spectrometry sequencing of immunoglobulin and amplification of variable genes of immunoglobulin light and heavy chains. **a** Purified IgG with neutralizing antibodies potential was denatured with heat, analyzed by SDS-PAGE, and stained with Coomassie Brilliant Blue. Cut at 55kD and 25kD, which are IgG heavy chain and light chain positions, respectively, and send to the Proteome Analysis Platform of the Peking University Medical Department and Health Analysis Center. **b** B cells from 15 COVID-19 convalescents were isolated from fresh or previously frozen PBMCs by immunomagnetic positive selection. Total RNA was extracted, then the cDNA was synthesized by 5′-RACE. BCR transcripts were amplificated with barcoded primers

Next, the three peptides and S protein were used to prepare affinity chromatography columns respectively. Purified IgG from the sera of 15 COVID-19 convalescent patients was used to sequentially bind to affinity chromatography columns of E1, E2-E3, E4, and S1 protein, respectively (Fig. 3b). Then, the neutralization activity of the eluted IgG that block SARS-CoV-2-S protein binding to ACE2-HEK293 was identified by using flow cytometry or luciferase experiments (Fig. 3c,d, Supplementary Fig. 1). Our results revealed that all IgG purified by three peptides displayed neutralizing activity. Interestingly, different individuals can selectively produce antibodies against three different epitope peptides. For example, the E1 and E2-specific IgG only showed in individuals 2 and 9, E2 and E3 specific IgG only showed in individuals 11 and 12, and E3 specific IgG only showed in individuals 6 and 7. Among them, the epitope peptide binding IgG from individuals 7, 8, and 9 showed strong neutralizing activity (Fig. 3c,d). In addition, we paid special attention to whether the results of neutralization activity of S protein-specific binding IgG are consistent with that of three epitope peptides. The results showed that the neutralization activity of S protein-specific binding IgG is consistent with that of epitope peptide-specific binding IgG in each individual (Fig. 3c,d), which suggested that the neutralizing epitopes of S protein are mainly concentrated in the four epitopes selected in this study, but not in other epitopes of S protein. Next, the variable region of neutralizing IgG that binding to S protein or three epitope peptides was analyzed by mass spectrographic analysis. The peptide information of the variable region of IgG will be mapped with the subsequent BCR sequence to determine the full-length sequence of each antibody variable region (Fig. 4a). These data indicate that the candidate epitopes can induce the body to produce specific antibodies with neutralizing activity, among which E1 and E4 epitopes are relatively conservative, and the neutralizing antibodies against E1 and E4 epitopes can also be used to block Omicron’s infection.

### Analysis of the total BCR repertoire of convalescents with COVID-19 of by 5′-RACE-related RT-PCR and PacBio sequencing

In order to obtain unbiased and entire sequence of variable region, 5′-RACE-related PCR was used in this study. In this method, when mRNA is reverse transcribed into cDNA, a certain nucleotide sequence is added to the 5′ end of cDNA as the upstream 5′ primer sequence, the downstream primer is complementary to the constant region of different heavy chain and light chain of BCR as the specific primers. Thus, it is possible to obtain all BCR transcripts. We have amplified the transcripts of variable region of Ig heavy chain and light chain respectively in B cells from 15 COVID-19 convalescent patients, which need to add a unique barcode to each PCR product and apply to high-throughput sequencing. Moreover, because the PCR product contains 5′ untranslated region of BCR, the length of the amplified DNA is more than 600 bp, which cannot be sequenced by the 250х250 next-generation sequencing platform we previously used, so PacBio sequencing technology was used in this study (Fig. 4b). By the 5′-RACE-related-PCR combined high throughput PacBio sequencing technology, we obtained 27,131 V(D) J sequences of Ig heavy chain and Ig light chain from 15 convalescents; among them, 365 V(D) J sequences only existed in 9 convalescents with neutralizing antibodies.

**Fig. 5:**
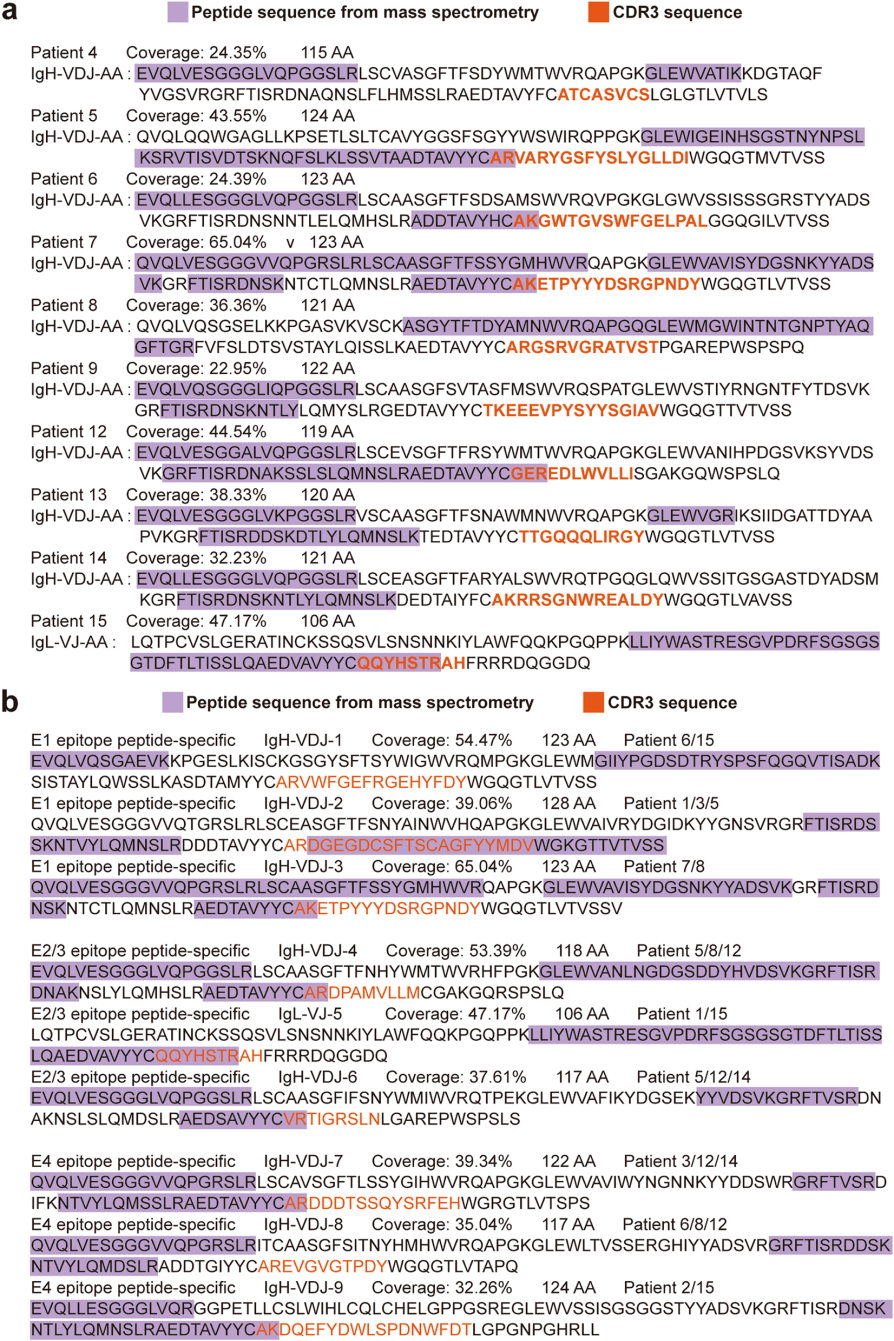
Match the mass spectrometry sequence with the sequencing sequence. **a** The mass spectrometry sequences were matched with the sequencing sequence from the same patient. The highly matched sequences that only exist in only one of the patients were shown. **b** The highly matched sequences that exist in different patients were shown

Subsequently, the peptide sequences of the variable region of neutralizing antibodies binding to E1, E2, E3, and S1 protein were used to map to the V_H_DJ_H_, V_κ_J_κ_ or V_λ_J_λ_ sequences of BCR in the same individual, and found that there are 10 of 15 convalescents showed one-to-one match between the peptide sequences and gene sequence (Fig. 5). Moreover, 55 sequences were shared by multiple individuals in COVID-19 convalescents with neutralizing antibodies but not exist in COVID-19 convalescents without neutralizing antibodies and healthy people (Fig. 5, Supplementary Fig. 2). These data showed that we have obtained the entire sequence of neutralizing antibodies.

### CDR3 sequences with potential neutralizing activity which can bind to S protein of SARS-CoV-2 and block SARS-CoV-2 infection

**Fig. 6:**
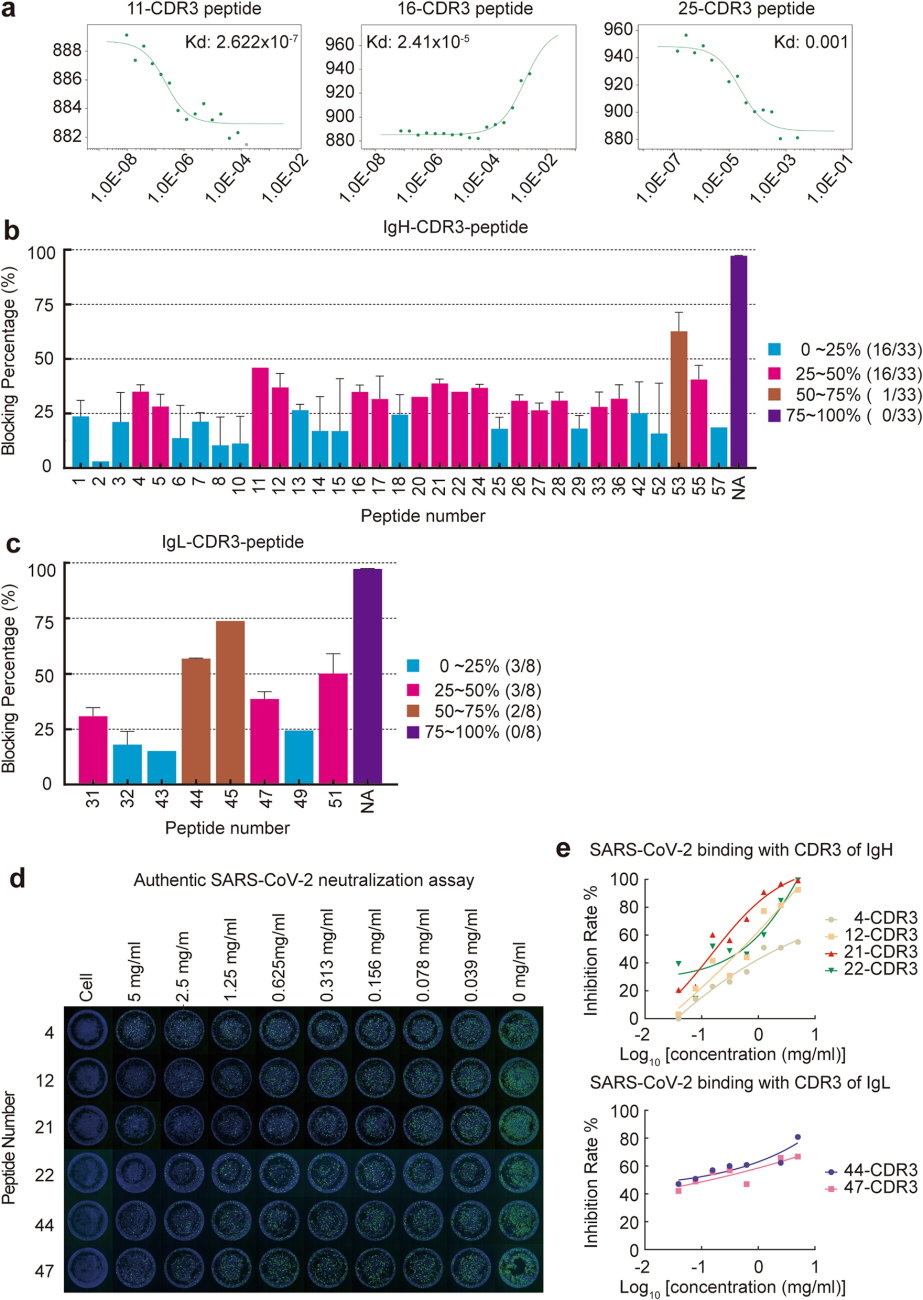
Neutralization ability of SARS-CoV-2-S1 epitope-specific CDR3 of IgG. **a** The binding ability of CDR3 peptides to S1 protein was analyzed by microscale thermophoresis (MST). CDR3 peptide 11, 16, and 25 were analyzed. **b**,**c** CDR3 peptides of these heavy chains and light chains with neutralizing potential were synthesized and detected their ability to block pseudoviruses by the method described in Fig. 2c. **c**, **d** The neutralization effect of CDR3 peptides (4, 12, 21, 22, 44, 47) to authentic SARS-CoV-2 was analyzed by IFA neutralization assay. The concentrations were from 5 mg/ml to 0 mg/ml. **e** The neutralization effect of CDR3 peptides (4, 12, 21, 22, 44, 47) to authentic SARS-CoV-2 was analyzed by FRNT assay. Peptide 4, 12, 21, 22 are CDR3 peptides of IgH. Peptide 44, 47 are CDR3 peptides of IgL

CDR3 of antibodies is a key domain in antigen-antibody interaction and determines the specificity of the antibody. To identify the neutralizing activity of candidate antibodies, we first synthesized 57 candidate CDR3 peptides of IgG with neutralizing activity (Supplementary Table 2). We first identified if CDR3 peptides can bind to S protein of SARS-CoV-2 by MST; clearly, the results revealed that several CDR3 peptides could bind to S protein of SARS-CoV-2 (Fig. 6a). Next, we further identified the neutralizing activities of all 57 candidate CDR3 peptides by pseudovirus assay and found that 50% of the CDR3 peptides had more than 30% blocking ability, and 3 CDR3 peptides showed more than 50% blocking ability, contained one CDR3 of heavy chain and 2 CDR3 of light chain (Fig. 6b,c). These data showed that the CDR3 peptide sorted by our method can successfully block the SARS-CoV-2 pseudovirus from infecting cells by binding to S protein.

### CDR3 peptides neutralized SARS-CoV-2 in vitro

Importantly, we further identified if the candidate CDR3 peptides can block SARS-CoV-2 infection in vitro. We established a SARS-CoV-2 infection model in Vero cells, neutralizing activity of 9 CDR3 peptides were selected and identified. Our results showed that 6 of 9 CDR3 peptides, including 4 CDR3 of IgH and 2 CDR3 of IgL, can prevent SARS-CoV-2 infection and showed a concentration-dependent manner. Neutralizing activity was assessed using two authentic virus neutralization assays, including IFA neutralization assay and focus reduction neutralization test (FRNT). Abs or peptide demonstrated obvious neutralizing activity against SARS-CoV-2 by IFA assay, including peptide 51# (IC_50_ of 51# peptide ranged from 1.25–2.5 mg/ml.), 53#, 21#, 22# 12#; while peptide 44#, 47#, 4# and16# had modestly lower neutralizing activity, 11# and 24# have no neutralizing activity. FRNT assay reconfirmed the neutralization effect (Fig. 6d,e). These data indicated that the CDR3 peptide sorted by our method can successfully block the SARS-CoV-2 infection in Vitro.

## Discussion

This study established a new strategy which can quickly and accurately identify the neutralizing antibody sequence. By this strategy, we sorted and synthesis several candidate CDR3 peptide and some of them (4#, 12#, 21#, 22#, 44#, 47#) showed neutralizing ability by both pseudovirus neutralizing assay and authentic virus neutralization assays.

It is known that there are many antigen epitopes on the S1 protein of SARS-CoV-2, and only the epitopes on RBD or adjacent RBD can induce neutralizing antibodies. Although most of the epitopes outside RBD can also induce specific antibodies. However, some of these antibodies only play a “bystander” effect, which may cause antibody-dependent enhancement (ADE) effect instead of neutralizing activity [14]. As expected, our results revealed that although almost all COVID-19 patients 2-week after recovery have high titers of IgG that can bind to S1 protein, only some of them have neutralizing activity. The observation further suggested that only a few B cells can recognize and produce neutralizing antibodies against the neutralizing epitope on S1 protein after SARS-CoV-2 infection. In other words, it is not easy to select neutralizing antibody sequences only from the BCR library that binds S1 protein.

In this study, we also found that, although the specific IgG against the potential neutralizing epitopes was found in all 15 COVID-19 convalescents, neutralization activity analysis clearly showed that only 9 convalescents detected neutralizing antibodies, which further indicated that even the IgG could bind to epitopes located on RBD or RBD adjacent regions, it does not necessarily have neutralizing activity. This result indicated that the B cell response to the same antigen is different among individuals due to different genetic factors. Our finding suggested that, only analysis the specific antibody titer against RBD of SARS-CoV-2 is not enough to reflect the degree of immune protection after virus infection or vaccination. Only relying on virus infection or vaccination will not induce all individuals to produce neutralizing antibodies.

In addition, the pandemic of Omicron-a new variant of SARS-CV-2 which showed increased transmissibility and immune invasion has spread worldwide. Existing vaccines and monoclonal antibodies seems effectiveness against Omicron due to its high mutation rates in the RBD, which make it difficult to be recognized by original antibodies [15]. Fortunately, however, our study showed that the E1 and E3 epitopes of the Omicron variant remained the same compared with wild-type SARS-CoV-2, suggesting that the neutralizing E1 and E3 epitopes we selected were very conservative and might be the candidate epitope that induce neutralizing antibody production in Omicron’s infection.

Therefore, it is important to acquire neutralizing antibodies against these conserved neutralizing epitope. At present, due to the limitation of protein mass spectrometry technology, the continuous full-length sequence of antibody variable regions cannot be obtained only by protein mass spectrometry. Thereby we designed a new scheme by mapping the peptide sequence of neutralizing antibodies obtained by protein mass spectrometry to the BCR repertoire obtained from the same individual. Moreover, we have made a more reasonable design to obtain a large number of full-length BCR libraries.

As we all know, the antibody repertoire in each individual presents infinite diversity to defend numerous antigens. At present, degenerate primers against V fragments combined with downstream primers complementary to J chain or C region were often used to amplify the variable region of antibody/BCR. This multiple-RT-PCR method cannot ensure that all of the V genes can be amplified equally. Thereby, in this study, we used the 5′-RACE-related-RT-PCR to replace the recently used multiple-RT-PCR method to avoid biased amplification of Ig variable region by multiple-RT-PCR.

As expected, using the novel strategy, we obtained a large number of full-length BCR library of COVID-19 convalescents. More importantly, by mapping the peptide sequence of neutralizing antibodies obtained by protein mass spectrometry to the BCR repertoire obtained from the same individual, we acquired numerous full-length V(D) J sequences of neutralizing antibodies. Moreover, using the CDR3 peptides of neutralizing antibodies, we have identified their neutralizing activity for SARS-CoV-2 infection. Here, we want to emphasize that the CDR3 of antibodies is a critical domain in antigen-antibody interaction and determines the specificity of the antibody. Compared with the entire antibody, the CDR3 peptide is easy to synthesis and avoids the ADE effect. In this study, we synthesized many candidate CDR3 peptides of BCR and carried out neutralization experiments. The results showed that more than 50% of the CDR3 peptides showed neutralization activity, demonstrating that CDR3 peptides can also prevent virus infection.

In conclusion, we have developed a new research scheme to quickly obtain the whole variable region sequences of antibodies which can be used to construct a SARS-CoV-2 specific antibody library. Moreover, the novel scheme will also be suitable for rapid screening neutralizing antibodies for other pathogenic microorganisms.

## Materials and methods

### Samples

Fifteen convalescent patients with COVID-19 2 weeks after recovery at the fifth Clinical Hospital of Peking University, during March 2020 were enrolled, ranged from ages 39 to 74. The clinical information of these patients was listed in Supplementary Table 1. This study and all the relevant experiments were approved by The fifth Clinical Hospital of Peking University Research Ethics Committee (reference numbers 04/023, 08/H0306/21, 08/H0308/176).

### Purification of IgG from sera

The plasma of convalescent patients with COVID-19 2 weeks after recovery and healthy donors were heat-inactivated (56 °C for 40 min) and incubated with Protein G Sepharose columns (GE Healthcare, Chicago, IL, USA) for 1 h at 4 °C, then allow the liquid phase to flow out, the Sepharose columns containing Protein G-IgG was washed with PBS. IgG were eluted from Protein G using 0.1 mol/L glycine (pH = 2.4). For buffer exchange to PBS, 3 kDa Amicon Ultra centrifugal filters (UFC900396, Merck-Millipore, Darmstadt, Germany) were used. Purified IgG concentration was measured using Nanodrop, and samples were stored at 4 °C.

### Virus and cell

The SARS-CoV-2 strains used in this research were isolated from COVID-19 patients in Guangzhou (NCBI, Accession numbers: MT123290), passaged and titered on Vero E6 cells- derived from an African Green monkey kidney which were grown in Dulbecco’s modified Eagle’s medium (DMEM, GIBCO, Grand Island, NY) supplemented with 10% fetal bovine serum (FBS). All work with SARS-CoV-2 was conducted in the Guangzhou Customs District Technology Center Biosafety Level 3 (BSL-3) Laboratory.

### ELISA analysis

ELISA plates were coated with S1 protein at 50 mM in carbonate coating buffer (pH = 9.6) at 4 °C overnight. After standard washing and blocking, diluted sera (from 1:100 to 1:30000) were applied to each well. After a 1 h incubation at 37 °C, plates were washed and incubated with 0.25 μg/ml goat anti-human IgG-conjugated with HRP (Southern Biotech, Birmingham, AL, USA) for 1 h at 37 °C. TMB was used as the substrate, and the reaction was ceased by 2 mol/L H_2_SO_4_. A microplate reader measured the absorbance at 450 nm.

### B cell epitope prediction

SARS-CoV-2 genome sequence and protein annotation were from Wuhan-Hu-1 isolate (GenBank accession number: MN908947). Linear B cell epitopes based on the spike protein antigen sequence characteristics using amino acid scales and hidden Markov models. Briefly, the methods of BepiPred 1.0 and BepiPred 2.0 were combined with default thresholds via the portal website (http://tools.iedb.org/bcell/). BepiPred 1.0 predicts the location of linear B-cell epitopes using a combination of a hidden Markov model and a propensity scale method (http://www.cbs.dtu.dk/services/BepiPred-1.0/). In contrast, BepiPred 2.0 predicts B-cell epitopes using a Random Forest algorithm trained on epitopes and non-epitope amino acids determined from crystal structures [16]. Because neutralizing antibodies were strongly expected, the epitopes in the receptor-binding motif (amino acids 438–506 in the full protein sequence) [3] and the adjacent region were paid more attention. The predicted epitopes were also checked the surface accessibility using the website ‘Emini Surface Accessibility Prediction’ method. We selected four linear B cell epitopes, including LFRKSNLKPFERDISTE (aa. 455–47, E1), YQAGSTPCNGV (aa. 473–483, E2), GFQPTNGVGY (aa. 496–505, E3), and QQFGRDIADTTDAVRDPQ (aa. 563–580, E4) for preferred peptide synthesis, the E1–3 are located on the receptor-binding domain (RBD), E4 is located on adjacent RBD. Then, the epitope peptides were synthesized by the Chinese Peptide Company, respectively. It is worth noting that since both E2 and E3 are short moreover, the distance between the two epitopes is very close. E2 and E3 are synthesized in series.

### Purification of epitope peptide-specific IgG

E1, E2-E3,E4 peptide and S protein (Purchase from OkayBio) were coupled to CNBr-activated Sepharose 4FF (17,098,101, GE Healthcare, Chicago, IL, USA) according to the manufacturer’s recommendation. Briefly, the preactivated Sepharose 4FF was suspended in 1 mM HCl for 30 min on ice and washed with cold coupling buffer (0.1 mol/L NaHCO3, 0.5 mol/L NaCl, pH 8.3). Then dissolve the peptide in coupling buffer and add to the washed sepharose and incubate overnight at 4 °C. Wash and resuspend the coupled gel in 0.1 mol/L Tris-HCl (pH 8.0) for 2 hours at room temperature to block unused activated sites. Then wash the gel six times with alternating 0.1 mol/L Tris, 0.5 mol/L NaCl, pH 8.0 and 0.1 mol/L NaAc, 0.5 mol/L NaCl, pH 4 buffers and then PBS. Next, the purified IgG from each COVID-19 patient was first incubated with E1-coupled Sepharose column overnight at 4 °C, then the suspension was transferred to E2/E3-coupled Sepharose column and incubation for 2 hours at room temperature (RT), the solution was collected and transferred to E4-coupled Sepharose column and incubation for 2 hours, RT. Finally, the suspension transferred to the S1-coupled Sepharose column. All columns as above were washed with PBS and eluted using 0.1 mol/L Glycine, pH 2.4 to obtain specific IgG for the E1, E2/3, E4, and other epitopes on S1 protein, respectively, and soon neutralized by 1 mol/L Tris, pH 8.0, and exchange to PBS by Amicon Ultra centrifugal filter.

### Pseudovirus neutralization assay

The pseudovirus neutralization assays were performed using hACE2-expressed HEK293 cell lines (purchase from PackGene Biotech). 5 × 10^4^/well cells were added to the well and cultured at 37 °C for 8 hours. The synthesized CDR3 peptides, IgG specifically bind to either E1-E4 or S1 protein, and sera were dissolved in DMEM complete medium and mixed with SARS-CoV-2 pseudovirus (purchased from Fubio Biological Technology Company, Pseudovirus-2019-nCOV) with a TCID50 of 5 × 10^4^ TU in a 1.5 ml Eppendorf tube with a 100 μL final volume (peptides at a final concentration 10 μg/μL, IgG at a final concentration 1 μg/μL, sera was diluted 160x) and incubated for 1 h at 37 °C. Negative control tube was supplied with 100 μL DMEM. Positive control tube was supplied with 100 μL DMEM containing SARS-CoV-2 pseudovirus. After the cells adhered to the wall, the supernatant was taken out from each well, and a 100 μL mixed solution of virus and peptides/antibodies was added to the well, and the two control groups were treated with the same operation. 48-well plate was cultured for 18 hours at 37 °C supplied with 5% CO_2_. The cells were directly washed with PBS and centrifuged at 3000 rpm, and the supernatant was discarded. 200 μL PBS was added, and the GFP-FITC was detected by flow cytometry. The neutralization efficiency of peptides/antibodies against SARS-CoV-2 pseudovirus was determined as [(Positive control MFI- experimental MFI)/(Positive control MFI- Negative control MFI)] × 100.

### SDS-PAGE and LC-mass/mass

Purified IgG with neutralizing antibodies potential was denatured with heat, analyzed by 12.5% SDS-polyacrylamide gel electrophoresis (PAGE), and stained with Coomassie Brilliant Blue. Cut at 55kD and 25kD, which are IgG heavy chain and light chain positions respectively, and send to the Proteome Analysis Platform of the Peking University Medical Department and Health Analysis Center.

### Enrichment of B cells from PBMC

B cells were isolated from fresh or previously frozen PBMCs by immunomagnetic positive selection according to the manufacturer’s protocol (EasySep™ Human CD19 Positive Selection Kit II, STEMCELL, Vancouver, Canada). Purified B cells were isolated and washed with PBS containing 2% (v/v) fetal bovine serum (FBS) and 1 mM EDTA.

### RNA extraction and cDNA synthesized by 5′-RACE

According to the manufacturer’s instructions for sorted B cells, total RNA was extracted using the RaPure Total RNA Micro Kit (Magen, Guangzhou, China), then the cDNA was synthesized by 5′-RACE using SMARTer® RACE 5′/3′ Kit (Takara Bio Inc., Shiga, Japan) and generated a complete cDNA copy with the additional specific sequence at the 5′ end.

### Amplification of BCR transcripts with barcoded primers

Nested PCR was used to amplify the variable regions of Ig. The upstream primer targeted an additional specific sequence from 5′-RACE, and the downstream primer targeted constant-region for *IGHG*, *IGHA*, *IGHM*, *IGHD*, *IGK*, and *IGL* in both of first-round and second-round PCR. Especially, barcodes were added to the second-round PCR primers that is convenient to distinguish BCRs from a different individual. PCR program for both rounds were: 5 cycles at 94 °C for the 30s, 5 cycles at 68 °C for 30s, 25 cycles at 72 °C for 3 minutes (first-round PCR) and 40 cycles at 94 °C for 30s, 68 °C for 30s, and 72 °C for 2 minutes (second-round PCR). The amplified DNA products were recovered from the agarose gel using a DNA Recovery Kit and sent to Novogene company for sequencing.

### Sequencing and barcode filtering

Sequencing libraries were prepared using PacBio sequencing. Raw reads were retained only if there are the sequences contained in the barcode. Constant region with highest sequence similarity was identified by matching to the reference constant region sequences from the IMGT database [17] and sequences were trimmed to give only the variable (VDJ) regions. Sequences with significant similarity to reference IGHV, D and J genes from the IMGT database using BLAST were retained [18]. Ig gene usages and sequence annotation were performed in IMGT V-QUEST, where repertoire differences were performed by custom scripts in Python.

### BCR repertoire analysis

Applying for an account in the IMGT database, login and use IMGT/HighV-QUEST (version 1.7.1) to submit the sequencing data to the IMGT database. Download the completed data, which were zip files, and decompress it into the folders. To get the CDR3 sequence and full length of variable region sequence corresponding to V(D) J usage in all samples by running scripts.

### BCR sequencing and mass spectrometric sequence alignment

The mass spectrometry peptide sequences of IgG that respectively bound to E1, E2-E3, E4, and S1 protein, were used to map to the variable region amino acid sequences obtained by BCR repertoire analysis from the same individual to obtain the complete sequence of IgG heavy chain and light chain variable region with neutralization potential. Moreover, we also try to find whether there were the same or similar IgG sequences with neutralization potential among different individuals. The analysis was completed with MaxQuant software.

### The binding ability of CDR3 peptides of IgG with neutralizing potential to S1 protein analyzed by microscale thermophoresis

Since antibodies recognize antigens mainly depend on the CDR3 region, we synthesize 57 CDR3 peptides of these heavy chains and light chains with neutralizing potential and verify their binding ability to S1 protein analyzed by microscale thermophoresis (MST). Briefly, single-cycle kinetics experiments with a Biacore T200 instrument (GE Healthcare) was used to analyze the binding of the S1 protein to the various CDR3 sequence. Purified S1 protein was first immobilized on a series S sensor chip protein A (GE Healthcare) at 800–1200 response units (RU) in PBS containing 0.02% sodium azide. One cell on the sensor chip was empty to serve as a blank. Then, a series of concentrations (i.e., 0.8, 4, 20, 100, and 500 nM) of soluble CDR3 peptide was injected in PBS at a flow rate of 60 μL/min. The sensor chip was regenerated using 10 mM Glycine-HCl (pH = 1.5) buffer. A 1:1 binding model was used to describe the experimental data. Due to conformational change in these cases, we fitted a two-state binding model that assumes two binding constants.

### Focus reduction neutralization test (FRNT)

FRNT assay was used for the evaluation of the Abs (peptide) neutralization effect. Vero E6 cells were seeded into a 96-well plate 1 day before infection. The next day, two-serially diluted Abs (peptide) and SARS-CoV-2 (80–120 FFU) were combined in DMEM (2% FBS) and incubated at 37 °C for 1 hour, then 50 μl mixtures were added into 96-well plate seeded with Vero E6 cells and incubated in 37 °C for 1 hour with rocking every 15 min. Then mixtures were removed and 100 μl MEM containing 1.2% Carboxymethylcellulose (1.2% CMC) was added. The medium was discarded after 24-hour post-infection, and the cell monolayer was fixed with 4% paraformaldehyde buffer at RT for 2 hours and permeabilized with 0.2% Triton X-100 for 20 min. Then the plates were sequentially stained with rabbit anti-SARS-CoV-2 N IgG (Cat. No. 40143-T62, Sino Biological Inc) and HRP-conjugated goat anti-rabbit IgG(H + L) (No.109–035-088, Jackson ImmunoResearch) at 37 °C for 1 hour respectively. The reactions were developed with KPL TrueBlue Peroxidase substrates and CTL ImmunoSpot S6 Ultra reader (Cellular Technology Ltd) was used to calculate the numbers of SARS-CoV-2 foci. The half-maximal inhibitory concentration (IC_50_) was determined by 50% focus reduction neutralization test titers (FRNT50) used to evaluate the potency of Abs in inhibiting SARS-CoV-2 replication.

### IFA neutralization assay

To determine whether Abs (peptide) could neutralize the infection of SARS-CoV-2, IFA neutralization assay was performed. Vero E6 cells were seeded into a 96-well plate 1 day before infection. Then, serial 2-fold diluted peptides were mixed with quantitative SARS-CoV-2 (MOI = 0.01) in microplates at 37 °C for 1 hour. Then the sample-virus mixture was transferred to the confluent cell monolayer in duplicate and incubated at a multiplicity of infection (MOI) of 0.01 at 37 °C for 24 h. After fixation with 4% paraformaldehyde, the monolayers were permeabilized with 0.2% triton X-100, followed by a 1 h incubation at 37 °C with the cross-reactive rabbit anti-SARS-CoV-2 N IgG (Sino Biological Inc) as the primary antibody. Then the cells were washed with PBS and incubated with Alexa Fluor 488 conjugated goat anti-rabbit IgG (Invitrogen) as the secondary antibody. Cells were washed twice with PBS and nuclei were stained with DAPI (Invitrogen, Germany) for 15 min at room temperature. Immunofluorescence was detected at 405 nm (DAPI) and 488 under Celigo Imaging Cytometer.

### Statistical analysis

All data were analyzed by normality and lognormality tests to identify whether the data belong to a normal distribution, which was decided by the *p*-value of the Shapiro-Wilk test. Unpaired t-test was used in the condition of normal distribution, or Mann-Whitney test was used in the non-normal distribution (* *p* < 0·05, ** *p* < 0·01, *** *p* < 0·005, **** *p* < 0·0001). These are all executed in GraphPad Prism.

## Supplementary Information

**Additional file 1.**

## Data Availability

The primary data of this study are available within the article and its Supplementary Figures. Source data are provided with this paper. All other data are available from the corresponding author upon reasonable request.
