# Supplementary material for: A new strategy: identification of specific antibodies for neutralizing epitope on SARS-CoV-2 S protein by LC-MS/MS combined with immune repertoire
Source: Mol Biomed. 2022 Jul 5;3:20. doi: 10.1186/s43556-022-00085-0 (PMC9253215; doi:10.1186/s43556-022-00085-0)
Supplement: Supplementary file 1 — Additional file 1. [file 43556_2022_85_MOESM1_ESM.pdf]

Title: A new strategy: identification of specific antibodies for neutralizing epitope on SARS-CoV-2 S protein by LC-MS/MS combined with immune repertoire

Meng Yu[1,2#], Zhu Zhu[1,2#], Yanqun Wang[3#], Pingzhang Wang[1,2#], Xiaodong Jia[4], Jie Wang[5], Lei Liu[6], Wanbing Liu[6], Yaqiong Zheng[6], Guomei Kou[6], Weiyan Xu[1,2], Jing Huang[1,2], Fengmin Lu[5], Xiajuan Zou[7], Shangen Zheng[6*], Yinying Lu[4*], Jincun Zhao[3*], Hui Dai[1,2*], Xiaoyan Qiu[1,2*]

[1] Department of Immunology, School of Basic Medical Sciences, Peking University, and NHC Key Laboratory of Medical Immunology (Peking University), Beijing, China.

[2] Key Laboratory of Molecular Immunology, Chinese Academy of Medical Sciences，Beijing, China.

[3] State Key Laboratory of Respiratory Disease, National Clinical Research Center for Respiratory Disease, Guangzhou Institute of Respiratory Health, the First Affiliated Hospital of Guangzhou Medical University, Guangdong, China.

[4] Department of Hepatology, Fifth Medical Center of Chinese PLA General Hospital, Beijing,China.

[5] Department of Microbiology and Infectious Disease Center, School of Basic Medical Sciences, Peking University Health Science Center, Beijing, China.

[6] Department of Transfusion Medicine, General Hospital of Central Theater Command of PLA, Wuhan, Hubei, China.

[7] Medical and Healthy Analysis Center, Peking University, Beijing,China.

[#] These authors contributed equally to this work.

*Correspondence to: Xiaoyan Qiu，Email: qiuxy@bjmu.edu.cn; Hui Dai, Email: daihui@bjmu.edu.cn; Jincun Zhao, Email: zhaojincun@gird.cn; Yinying Lu, Email: luyinying1973@163.com; Shangen Zheng, Email: sxkzsg@sina.com

Materials and Methods

Samples

Fifteen convalescent patients with COVID-19 2 weeks after recovery at the fifth Clinical Hospital of Peking University, during March 2020 were enrolled, ranged from ages 39 to 74. The clinical information of these patients was listed in Supplementary Table 1. This study and all the relevant experiments were approved by The fifth Clinical Hospital of Peking University Research Ethics Committee (reference numbers 04/023, 08/H0306/21, 08/H0308/176).

Purification of IgG from sera

The plasma of convalescent patients with COVID-19 2 weeks after recovery and healthy donors were heat-inactivated (56 °C for 40 min) and incubated with Protein G Sepharose columns (GE Healthcare, Chicago, IL, USA) for one hour at 4 °C, then allow the liquid phase to flow out, the Sepharose columns containing Protein G-IgG was washed with PBS. IgG were eluted from Protein G using 0.1 mol/L glycine (pH=2.4). For buffer exchange to PBS, 3 kDa Amicon Ultra centrifugal filters (UFC900396, Merck-Millipore, Darmstadt, Germany) were used. Purified IgG concentration was measured using Nanodrop, and samples were stored at 4 °C.

Virus and cell

The SARS-CoV-2 strains used in this research were isolated from COVID-19 patients in Guangzhou (NCBI, Accession numbers: MT123290), passaged and titered on Vero E6 cells-derived from an African Green monkey kidney which were grown in Dulbecco's modified Eagle's medium (DMEM, GIBCO, Grand Island, NY) supplemented with 10% fetal bovine serum (FBS). All work with SARS-CoV-2 was conducted in the Guangzhou Customs District Technology Center Biosafety Level 3 (BSL-3) Laboratory.

ELISA analysis

ELISA plates were coated with S1 protein at 50mM in carbonate coating buffer (pH=9.6) at 4 °C overnight. After standard washing and blocking, diluted sera (from 1:100 to 1:30000) were applied to each well. After a 1h incubation at 37 °C, plates were washed and incubated with 0.25 μg/ml goat anti-human IgG-conjugated with HRP (Southern Biotech, Birmingham, AL, USA) for 1h at 37 °C. TMB was used as the substrate, and the reaction was ceased by 2 mol/L H_2SO_4. A microplate reader measured the absorbance at 450nm.

B Cell Epitope Prediction

SARS-CoV-2 genome sequence and protein annotation were from Wuhan-Hu-1 isolate (GenBank accession number: MN908947). Linear B cell epitopes based on the spike protein antigen sequence characteristics using amino acid scales and hidden Markov models. Briefly, the methods of BepiPred 1.0 and BepiPred 2.0 were combined with default thresholds via the portal website (http://tools.iedb.org/bcell/). BepiPred 1.0 predicts the location of linear B-cell epitopes using a combination of a hidden Markov model and a propensity scale method (http://www.cbs.dtu.dk/services/BepiPred-1.0/). In contrast, BepiPred 2.0 predicts B-cell epitopes using a Random Forest algorithm trained on epitopes and non-epitope amino acids determined from crystal structures[17]. Because neutralizing antibodies were strongly expected, the epitopes in the receptor-binding motif (amino acids 438-506 in the full protein sequence)[18] and the adjacent region were paid more attention. The predicted epitopes were also checked the surface accessibility using the website 'Emini Surface Accessibility Prediction' method. We selected four linear B cell epitopes, including LFRKSNLKPFERDISTE (aa. 455-47, E1), YQAGSTPCNGV (aa. 473-483, E2), GFQPTNGVGY (aa. 496-505, E3), and QQFGRDIADTTDAVRDPQ (aa. 563-580, E4) for preferred peptide synthesis, the E1-3 are located on the receptor-binding domain (RBD), E4 is located on adjacent RBD. Then, the epitope peptides were synthesized by the Chinese Peptide Company， respectively. It is worth noting that since both E2 and E3 are short moreover, the distance between the two epitopes is very close. E2 and E3 are synthesized in series.

Purification of epitope peptide-specific IgG

E1, E2-E3, E4 peptide and S protein (Purchase from OkayBio) were coupled to CNBr-activated Sepharose 4FF (17098101, GE Healthcare, Chicago, IL, USA) according to the manufacturer's recommendation. Briefly, the preactivated Sepharose 4FF was suspended in 1mM HCl for 30 min on ice and washed with cold coupling buffer (0.1 mol/L NaHCO3, 0.5 mol/L NaCl, pH 8.3). Then dissolve the peptide in coupling buffer and add to the washed sepharose and incubate overnight at 4 °C. Wash and resuspend the coupled gel in 0.1mol/L Tris-HCl (pH 8.0) for 2 hours at room temperature to block unused activated sites. Then wash the gel six times with alternating 0.1 mol/L Tris, 0.5 mol/L NaCl, pH 8.0 and 0.1 mol/L NaAc, 0.5 mol/L NaCl, pH 4 buffers and then PBS. Next, the purified IgG from each COVID-19 patient

was first incubated with E1-coupled Sepharose column overnight at 4 °C, then the suspension was transferred to E2/E3-coupled Sepharose column and incubation for 2 hours at room temperature (RT), the solution was collected and transferred to E4-coupled Sepharose column and incubation for 2 hours, RT. Finally, the suspension transferred to the S1-coupled Sepharose column. All columns as above were washed with PBS and eluted using 0.1 mol/L Glycine, pH 2.4 to obtain specific IgG for the E1, E2/3, E4, and other epitopes on S1 protein, respectively, and soon neutralized by 1 mol/L Tris, pH 8.0, and exchange to PBS by Amicon Ultra centrifugal filter.

Pseudovirus neutralization assay

The pseudovirus neutralization assays were performed using hACE2-expressed HEK293 cell lines (purchase from PackGene Biotech). 5×10^4/well cells were added to the well and cultured at 37 °C for 8 hours. The synthesized CDR3 peptides, IgG specifically bind to either E1-E4 or S1 protein, and sera were dissolved in DMEM complete medium and mixed with SARS-CoV-2 pseudovirus (purchased from Fubio Biological Technology Company, Pseudovirus-2019-nCOV) with a TCID50 of 5×10^4 TU in a 1.5 ml Eppendorf tube with a 100μL final volume (peptides at a final concentration 10 μg/μL, IgG at a final concentration 1 μg/μL, sera was diluted 160x) and incubated for one hour at 37 °C. Negative control tube was supplied with 100 μL DMEM. Positive control tube was supplied with 100 μL DMEM containing SARS-CoV-2 pseudovirus. After the cells adhered to the wall, the supernatant was taken out from each well, and a 100 μL mixed solution of virus and peptides/antibodies was added to the well, and the two control groups were treated with the same operation. 48-well plate was cultured for 18 hours at 37 °C supplied with 5% CO_2. The cells were directly washed with PBS and centrifuged at 3000rpm, and the supernatant was discarded. 200 μL PBS was added, and the GFP-FITC was detected by flow cytometry. The neutralization efficiency of peptides/antibodies against SARS-CoV-2 pseudovirus was determined as [(Positive control MFI- experimental MFI)/(Positive control MFI- Negative control MFI)] × 100.

SDS-PAGE and LC-Mass/Mass

Purified IgG with neutralizing antibodies potential was denatured with heat, analyzed by 12.5% SDS-polyacrylamide gel electrophoresis (PAGE), and stained with Coomassie Brilliant Blue. Cut at 55kD and 25kD, which are IgG heavy chain and light chain positions respectively,

and send to the Proteome Analysis Platform of the Peking University Medical Department and Health Analysis Center.

Enrichment of B cells from PBMC

B cells were isolated from fresh or previously frozen PBMCs by immunomagnetic positive selection according to the manufacturer's protocol (EasySep™ Human CD19 Positive Selection Kit II, STEMCELL, Vancouver, Canada). Purified B cells were isolated and washed with PBS containing 2% (v/v) fetal bovine serum (FBS) and 1 mM EDTA.

RNA extraction and cDNA synthesized by 5'-RACE

According to the manufacturer's instructions for sorted B cells, total RNA was extracted using the RaPure Total RNA Micro Kit (Magen, Guangzhou, China), then the cDNA was synthesized by 5'-RACE using SMARTer® RACE 5'/3' Kit (Takara Bio Inc., Shiga, Japan) and generated a complete cDNA copy with the additional specific sequence at the 5' end.

Amplification of BCR transcripts with barcoded primers

Nested PCR was used to amplify the variable regions of Ig. The upstream primer targeted an additional specific sequence from 5'-RACE, and the downstream primer targeted constant-region for *IGHG*, *IGHA*, *IGHM*, *IGHD*, *IGK*, and *IGL* in both of first-round and second-round PCR. Especially, barcodes were added to the second-round PCR primers that is convenient to distinguish BCRs from a different individual. PCR program for both rounds were: 5 cycles at 94 °C for the 30s, 5 cycles at 68 °C for 30s, 25 cycles at 72 °C for 3 minutes (first-round PCR) and 40 cycles at 94 °C for 30s, 68 °C for 30s, and 72 °C for 2 minutes (second-round PCR). The amplified DNA products were recovered from the agarose gel using a DNA Recovery Kit and sent to Novogene company for sequencing.

Sequencing and barcode filtering

Sequencing libraries were prepared using PacBio sequencing. Raw reads were retained only if there are the sequences contained in the barcode. Constant region with highest sequence similarity was identified by matching to the reference constant region sequences from the IMGT database[19] and sequences were trimmed to give only the variable (VDJ) regions. Sequences with significant similarity to reference IGHV, D and J genes from the IMGT database using BLAST were retained[20]. Ig gene usages and sequence annotation were performed in IMGT V-QUEST, where repertoire differences were performed by custom scripts

in Python.

BCR repertoire analysis

Applying for an account in the IMGT database, login and use IMGT/HighV-QUEST (version 1.7.1) to submit the sequencing data to the IMGT database. Download the completed data, which were zip files, and decompress it into the folders. To get the CDR3 sequence and full length of variable region sequence corresponding to V(D)J usage in all samples by running scripts.

BCR sequencing and Mass spectrometric sequence alignment

The mass spectrometry peptide sequences of IgG that respectively bound to E1, E2-E3, E4, and S1 protein, were used to map to the variable region amino acid sequences obtained by BCR repertoire analysis from the same individual to obtain the complete sequence of IgG heavy chain and light chain variable region with neutralization potential. Moreover, we also try to find whether there were the same or similar IgG sequences with neutralization potential among different individuals. The analysis was completed with MaxQuant software.

The binding ability of CDR3 peptides of IgG with neutralizing potential to S1 protein analyzed by microscale thermophoresis

Since antibodies recognize antigens mainly depend on the CDR3 region, we synthesize 57 CDR3 peptides of these heavy chains and light chains with neutralizing potential and verify their binding ability to S1 protein analyzed by microscale thermophoresis (MST). Briefly, single-cycle kinetics experiments with a Biacore T200 instrument (GE Healthcare) was used to analyze the binding of the S1 protein to the various CDR3 sequence. Purified S1 protein was first immobilized on a series S sensor chip protein A (GE Healthcare) at 800-1200 response units (RU) in PBS containing 0.02% sodium azide. One cell on the sensor chip was empty to serve as a blank. Then, a series of concentrations (i.e., 0.8, 4, 20, 100, and 500 nM) of soluble CDR3 peptide was injected in PBS at a flow rate of 60 µL/min. The sensor chip was regenerated using 10 mM Glycine-HCl (pH=1.5) buffer. A 1:1 binding model was used to describe the experimental data. Due to conformational change in these cases, we fitted a two-state binding model that assumes two binding constants.

Focus reduction neutralization test (FRNT)

FRNT assay was used for the evaluation of the Abs (peptide) neutralization effect. Vero

E6 cells were seeded into a 96-well plate one day before infection. The next day, two-serially diluted Abs (peptide) and SARS-CoV-2 (80-120 FFU) were combined in DMEM (2% FBS) and incubated at 37 °C for 1 hour, then 50μl mixtures were added into 96-well plate seeded with Vero E6 cells and incubated in 37 °C for 1 hour with rocking every 15 min. Then mixtures were removed and 100 μl MEM containing 1.2% Carboxymethylcellulose (1.2% CMC) was added. The medium was discarded after 24-hour post-infection, and the cell monolayer was fixed with 4% paraformaldehyde buffer at RT for 2 hours and permeabilized with 0.2% Triton X-100 for 20 min. Then the plates were sequentially stained with rabbit anti-SARS-CoV-2 N IgG (Cat. No. 40143-T62, Sino Biological Inc) and HRP-conjugated goat anti-rabbit IgG(H+L) (No.109-035-088, Jackson ImmunoResearch) at 37 °C for 1 hour respectively. The reactions were developed with KPL TrueBlue Peroxidase substrates and CTL ImmunoSpot S6 Ultra reader (Cellular Technology Ltd) was used to calculate the numbers of SARS-CoV-2 foci. The half-maximal inhibitory concentration (IC_{50}) was determined by 50% focus reduction neutralization test titers (FRNT50) used to evaluate the potency of Abs in inhibiting SARS-CoV-2 replication.

IFA neutralization assay

To determine whether Abs (peptide) could neutralize the infection of SARS-CoV-2, IFA neutralization assay was performed. Vero E6 cells were seeded into a 96-well plate one day before infection. Then, serial 2-fold diluted peptides were mixed with quantitative SARS-CoV-2 (MOI=0.01) in microplates at 37 °C for 1 hour. Then the sample-virus mixture was transferred to the confluent cell monolayer in duplicate and incubated at a multiplicity of infection (MOI) of 0.01 at 37 °C for 24h. After fixation with 4% paraformaldehyde, the monolayers were permeabilized with 0.2% triton X-100, followed by a 1 h incubation at 37 °C with the cross-reactive rabbit anti-SARS-CoV-2 N IgG (Sino Biological Inc) as the primary antibody. Then the cells were washed with PBS and incubated with Alexa Fluor 488 conjugated goat anti-rabbit IgG (Invitrogen) as the secondary antibody. Cells were washed twice with PBS and nuclei were stained with DAPI (Invitrogen, Germany) for 15 min at room temperature. Immunofluorescence was detected at 405 nm (DAPI) and 488 under Celigo Imaging Cytometer.

Statistical Analysis

All data were analyzed by normality and lognormality tests to identify whether the data

belong to a normal distribution, which was decided by the p-value of the Shapiro-Wilk test. Unpaired t-test was used in the condition of normal distribution, or Mann-Whitney test was used in the non-normal distribution (* $p<0{\cdot}05$, ** $p<0{\cdot}01$, *** $p<0{\cdot}005$, **** $p<0{\cdot}0001$). These are all executed in GraphPad Prism.

Supplementary tables and Figures

Supplementary Figure 1. Ability of blocking pseudovirus in purified antibodies

a. E1, E2-E3, E4, and S1 protein-specific IgGs purified by affinity column from 3 healthy subjects (HS) and 15 COVID-19 convalescents have been detected the ability to block pseudovirus by flow cytometry as described in Fig 2c. B. E1, E2-E3, E4, and S1 protein-specific IgGs purified by affinity column from healthy subjects (HS) and COVID-19 convalescents have been detected the ability to block pseudovirus by luciferase assay.

Supplementary Figure 2. Distribution of highly matched sequences

a. The frequency of highly matched sequences that only exist in only one of the patients was shown

b. The frequency of highly matched sequences that exist in different patients was shown

Supplementary Table 1. The clinical information of COVID-19 patients with 2-week convalescent

Supplementary Table 2. Amino acid sequence of candidate CDR3 peptide.

Supplementary Table 3. Amino acid mutation of Omicron variants in our candidate epitope.

Supplementary Table 1. The clinical information of COVID-19 patients with 2-week convalescent

Patient No.	1	3	4	5	6	7	8	9	10	11	12	13	14	15
severity	Moderate	Moderate	Moderate	Severity	Severity	Moderate	Severity	Severity	Severity	Critical	Critical	Moderate	Moderate	Critical
gender	M	F	M	M	M	F	F	F	M	M	M	F	F	F
Age	66	65	64	67	39	69	74	60	67	78	74	39	62	67
White blood cells (10^9 /liter)	3.68	6.74	3.68	5.32	4.33	3.43	11.22	3.21	5.32	3.69	10.45	4.22	5.25	9.59
Percentage of neutrophil	54.3	69.8	60.4	77.6	81.1	71.7	77.6	34.1	77.6	82.1	94.1	45.5	67.6	94
Numbers of neutrophils (10^9 /liter)	2	4.7	3.4	4.12	3.51	2.46	8.71	1.09	4.12	3.03	9.83	2.15	3.54	9.01
Percentage of lymphocytes	37.8	23.1	31.1	11.6	16.9	20.6	15.9	41.5	11.6	11.2	3.3	46.6	23.6	3.4
Numbers of lymphocytes (10^9 /liter)	1.39	1.56	1.75	0.62	0.73	0.71	1.78	1.68	0.62	0.41	0.35	1.77	1.24	0.33
Percentage of monocytes	6.5	6.1	5.3	7.2	1.6	6.4	6.4	10.4	7.2	5.7	0.35	6.2	7.1	2.5
Numbers of monocytes (10^9 /liter)	0.24	0.41	0.3	0.38	0.07	0.22	0.72	0.54	0.38	0.21	0.26	0.26	0.37	0.24
Red blood cells (10^{12}/liter)	4.81	4.09	3.97	3.74	3.49	3.58	3.42	3.68	3.74	3.3	3.22	2.99	3.03	2.77
Hemoglobin (g/liter)	133	124	120	125	97	109	106	112	125	98	98	86	99	86
History of drink	No	No	Yes	Yes	Yes	No	No	No	Yes	Yes	No	No	No	No
History of smoke	No	No	Yes	No	No	No	No	No	No	No	No	No	No	No

Supplementary Table 2. Amino acid sequence of candidate CDR3 peptide.

No.	CDR3 sequence	Accession	V(D)J usage
1	ARGWHDYIWGSPVGGAFDI	7S3H	IGHV5-51*01,IGHD3-16*01,IGHJ3*02
2	ARGIKCDTTSCHFYMDV	12S2H	IGHV4-34*01,IGHD2-2*01,IGHJ6*03
3	AKSRGYGDFFL	7S3H	IGHV4-59*01,IGHD3-10*01,IGHJ4*02
4	VRYSPEPAVLITEGYFD	12S2H	IGHV4-59*01,IGHD2-2*01,IGHJ4*02
5	VRRRERALWT	7S3H	IGHV3-72*01,IGHD5-24*01,IGHJ6*02
6	VRGGGIGVALT	12S2H	IGHV3-33*01,IGHD6-19*01,IGHJ4*02
7	VREAAWNNYQ	12S2H	IGHV3-7*01,IGHD1-1*01,IGHJ1*01
8	ATVGYCSSPSCYNIFHMDV	12S3H	IGHV1-24*01,IGHD2-2*01,IGHJ6*03
9	ARLLVVTRSKYFD	12S2H	IGHV3-53*01,IGHD4-23*01,IGHJ4*02
10	ARGHFVVEPAALWKRYGDFYFDS	7S3H	IGHV4-34*02,IGHD2-2*01,IGHJ4*02
11	ARESLYDFDGSGYVDALDV	15S2H	IGHV3-48*03,IGHD3-22*01,IGHJ3*01
12	ARERADDYSNYCWFDP	12S3H	IGHV1-69*06,IGHD4-11*01,IGHJ5*02
13	AREPRPYYDILTGYSSPKRTLT	8S1H	IGHV3-48*03,IGHD3-9*01,IGHJ4*02
14	ARDRRYDFWSGGDFD	12S2H	IGHV3-30*09,IGHD3-3*01,IGHJ4*02
15	ARDREGVVPSPVLGLGPYYEYHAVEV	7S3H	IGHV4-34*01,IGHD3-10*01,IGHJ6*02
16	ARDIVVPAAMGEIGGWFDP	8S1H	IGHV3-48*03,IGHD2-2*01,IGHJ5*02
17	ARDGADGDTWEFYYNGMDV	12S2H	IGHV3-33*01,IGHD2-2*03,IGHJ6*02
18	ARAQRSGDYFYGMDV	12S2H	IGHV3-66*02,IGHD2-15*01,IGHJ6*02
19	YYCAQGMWLDR	12S3H	IGHV3-23*03,IGHD6-19*01,IGHJ5*02
20	AKVKGQGYTDWRVGNFDY	12S2H	IGHV3-30*03,IGHD5-24*01,IGHJ4*03
21	AKDFRQYCSSTTCYDLDY	7S0H	IGHV3-9*01,IGHD2-2*01,IGHJ4*02
22	TTDRYQLLWAG	12S2H	IGHV3-15*01,IGHD2-2*01,IGHJ4*02
23	TTGWFWSHYKKNDY	6S3H	IGHV3-15*01,IGHD3-3*01,IGHJ4*02
24	ARDVSSLYGGNSDDY	12S2H	IGHV1-46*01,IGHD4-23*01,IGHJ4*02
25	ARDLGLPSNVGRWYMGDLGY	12S2H	IGHV1-18*01,IGHD6-25*01,IGHJ4*02
26	ARDQRGSSWYGGEF	8S0H	IGHV1-18*01,IGHD6-13*01,IGHJ4*02
27	AREAPLPRVTVSSSSPFDY	12S3H	IGHV3-21*06,IGHD2-2*01,IGHJ4*02
28	ARGSGFSYASLQEHYGLDV	12S3H	IGHV3-30-3*01,IGHD5-18*01,IGHJ6*02

No.	CDR3 sequence	Accession	V(D)J usage
29	ARPYQSSGWYFEY	12S2H	IGHV1-46*01,IGHD6-19*01,IGHJ4*02
30	ASSYYMIVVPPACPTTTTTVWT	7S0H	IGHV3-66*01,IGHD3-22*01,IGHJ6*02
31	CSRDTSGNHLV	7S3L	IGLV3-19*01,,IGLJ3*02
32	DYYCQSYDNMRV	7S3L	IGLV1-40*01,,IGLJ3*02
33	TTNLGSRGYYHVVMDYFD	12S2H	IGHV3-15*01,IGHD3-22*01,IGHJ1*01
34	TYYCLQITFTR	12S3L	IGLV4-60*03,,IGLJ2*01
35	TYYCQQSFGTPT	7S0L	IGKV1-39*01,,IGKJ4*01
36	YYCANSGGLG	12S3H	IGHV3-23*01,IGHD2-15*01,IGHJ4*02
37	YYCARATGDWYFDL	8S1L2	IGHV3-13*05,IGHD7-27*01,IGHJ2*01
38	YYCARDSSNWYPNS	7S0H	IGHV3-53*01,IGHD6-13*01,IGHJ4*01
39	YYCASYAGDNTFV	8S1L2	IGLV2-8*01,,IGLJ2*01
40	YYCGTWDSTLSAL	8S0L	IGLV1-51*01,,IGLJ3*02
41	YYCHQSSGLPQT	7S3L	IGLV3-21*04,,IGLJ3*02
42	YYCLRGHYDFDIF	12S2H	IGHV3-15*04,IGHD3-9*01,IGHJ4*02
43	YYCQQRSNWPPLFT	12S3L	IGKV3-11*01,,IGKJ3*01
44	YYCQQYDNLSGT	7S3L	IGKV1-33*01,,IGKJ3*01
45	YYCQQYITYPFT	7S0L	IGKV1-5*03,,IGKJ2*01
46	YYCQQYYSTPRLY	6S3L	IGKV4-1*01,,IGKJ4*01
47	YYCQSYDSSNVV	8S0L	IGLV6-57*03,,IGLJ2*01
48	YYCQQYHSTRAH	15S2L	IGLV3-9*01,,IGLJ2*01
49	YYCQVWDNNAAV	12S3L	IGLV3-9*01,,IGLJ3*02
50	ARGLKWGQGAQ	12S2H	IGHV3-48*03,,IGHJ4*02
51	EAWDVDNRVFGGGTR	7S0L	IGKV4-1*01,,IGKJ1*01
52	SKDTFDYWGHGT	12S2H	IGHV3-43*01,,IGHJ4*01
53	VRERISYFDHWGQG	12S2H	IGHV4-31*03,IGHD6-6*01,IGHJ4*02
54	VRYSPEPAVLITEGYFD	12S3H	IGHV4-59*01,IGHD2-2*01,IGHJ4*02
55	ARGRGVGTVATPPLDY	12S3H	IGHV1-2*02,IGHD4-23*01,IGHJ4*02
56	ATDGRRGYSGYD	12S3H	IGHV1-24*01,IGHD5-12*01,IGHJ4*02
57	GRVAENRTGYYYGDY	12S3H	IGHV3-74*01,IGHD3-9*01,IGHJ4*02

Supplementary Table 3. Amino acid mutation of Omicron variants in our candidate epitope.

**Omicron's variants**	**E1（455-471）**	**E2/3（473-483/496-505）**	**E4（563-580）**
BA.1	None	S477N, T478K, G496S, Q498R, N501Y, Y505H	None
BA.2	None	S477N, T478K, Q498R, N501Y, Y505H	None
BA.2.12.1	None	S477N, T478K, Q498R, N501Y, Y505H	None
BA.3	None	S477N, T478K, Q498R, N501Y, Y505H	None
BA.4	None	S477N, T478K, Q498R, N501Y, Y505H	None
BA.5	None	S477N, T478K, Q498R, N501Y, Y505H	None

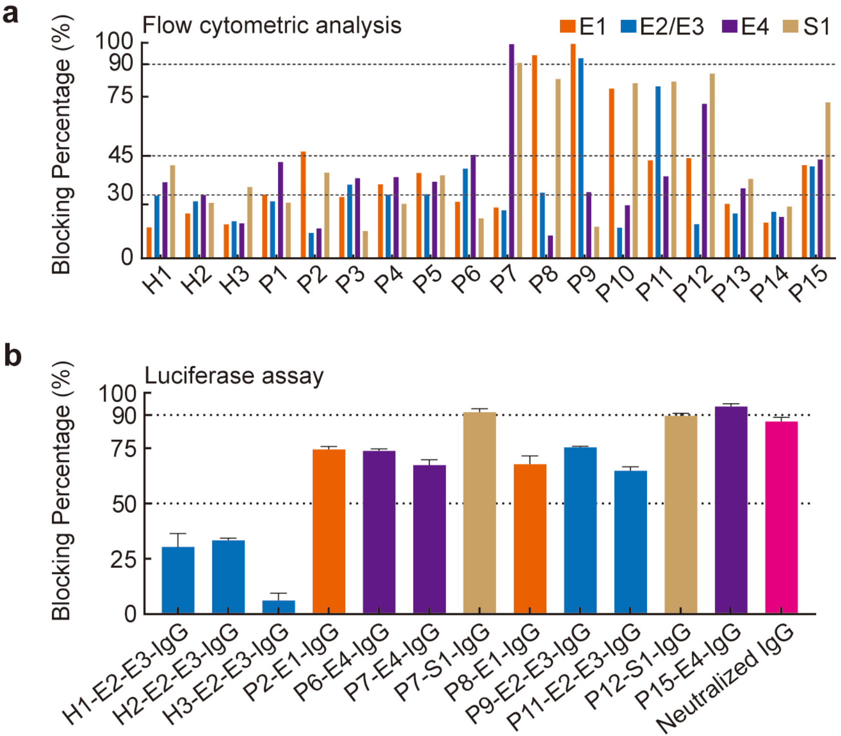
a
Flow cytometric analysis
E1
E2/E3
E4
S1
Blocking Percentage (%)
100
90
75
45
30
0
H1
H2
H3
P1
P2
P3
P4
P5
P6
P7
P8
P9
P10
P11
P12
P13
P14
P15
b
Luciferase assay
Blocking Percentage (%)
100
90
75
50
25
0
H1-E2-E3-IgG
H2-E2-E3-IgG
H3-E2-E3-IgG
P2-E1-IgG
P6-E4-IgG
P7-E4-IgG
P7-S1-IgG
P8-E1-IgG
P9-E2-E3-IgG
P11-E2-E3-IgG
P12-S1-IgG
P15-E4-IgG
Neutralized IgG

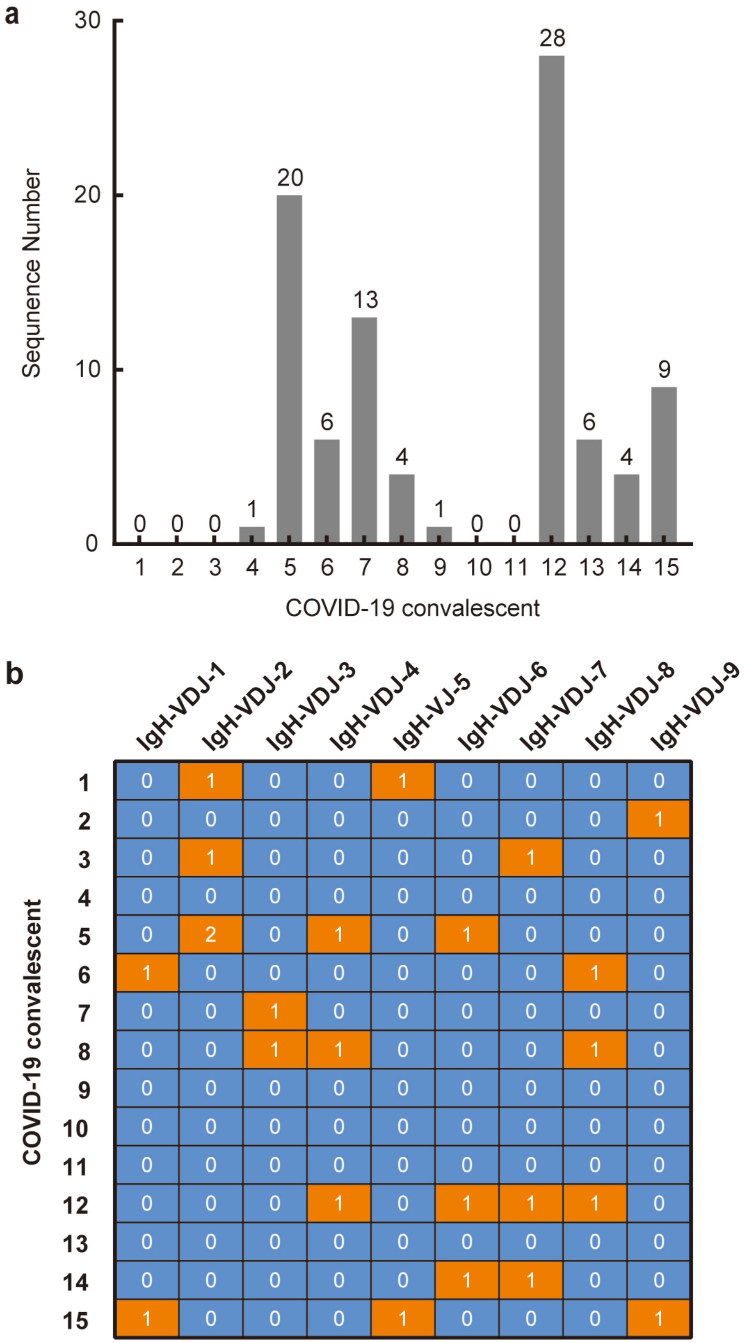
a
Sequnence Number
0
10
20
30
0 0 0 1 20 6 13 4 1 0 0 28 6 4 9
1 2 3 4 5 6 7 8 9 10 11 12 13 14 15
COVID-19 convalescent
b
IgH-VDJ-1 IgH-VDJ-2 IgH-VDJ-3 IgH-VDJ-4 IgH-VJ-5 IgH-VDJ-6 IgH-VDJ-7 IgH-VDJ-8 IgH-VDJ-9
COVID-19 convalescent
1 0 1 0 0 1 0 0 0 0
2 0 0 0 0 0 0 0 0 1
3 0 1 0 0 0 0 1 0 0
4 0 0 0 0 0 0 0 0 0
5 0 2 0 1 0 1 0 0 0
6 1 0 0 0 0 0 0 1 0
7 0 0 1 0 0 0 0 0 0
8 0 0 1 1 0 0 0 1 0
9 0 0 0 0 0 0 0 0 0
10 0 0 0 0 0 0 0 0 0
11 0 0 0 0 0 0 0 0 0
12 0 0 0 1 0 1 1 1 0
13 0 0 0 0 0 0 0 0 0
14 0 0 0 0 0 1 1 0 0
15 1 0 0 0 1 0 0 0 1
